# Viral diversity in urban feline swab samples recovered using metagenomics

**DOI:** 10.1128/mra.00131-25

**Published:** 2025-07-17

**Authors:** Taehyung Kwon, Brett Youtsey, Nelson Ruth, Jeanne M. Fair, Cheryl D. Gleasner, Migun Shakya, Azra Marghoob, Andrew W. Bartlow

**Affiliations:** 1 Biochemistry and Biotechnology group (B-TEK), Bioscience Division, Los Alamos National Laboratory, Los Alamos, New Mexico, USA; 2 Genomics and Bioanalytics group (B-GEN), Bioscience Division, Los Alamos National Laboratory, Los Alamos, New Mexico, USA; 3 Companions Animal Hospital, Merrick, New York, USA; Katholieke Universiteit Leuven, Leuven, Belgium

**Keywords:** metagenomics, cat, virus, feline foamy virus, felid alphaherpesvirus

## Abstract

The relationship between domestic cats and humans may facilitate transmission of viruses between the two hosts. Metagenomic analyses of 70 urban feline swab samples identified six high-quality draft genomes from felid alphaherpesvirus 1 and feline foamy virus.

## ANNOUNCEMENT

Domestic cats (*Felis catus*) are in close contact with humans as household pets and can carry various pathogens. Spillover events can occur from cats to humans, but reverse zoonotic transmission from humans to cats is also reported (1, 2). Viral diversity in domestic cats should be actively monitored using unbiased diagnostic tools, such as metagenomics.

We collected rectal and throat swabs from Companions Animal Clinic in Merrick, NY, between April and August 2020 from 33 domesticated cats (31 household cats, two strays) and collected two rectal and throat swabs from one cat visiting the clinic twice, totaling 70 samples. Swabs were placed in Viral Transport Media (VTM; ThermoFisher Scientific), frozen, and shipped to Los Alamos, NM for sequencing. Total nucleic acid was extracted from 140 µL VTM using the Qiagen QIAmp Viral RNA Mini Kit (Cat. #52904) and eluted in 60 µL AVE buffer. rRNAs were depleted from 50 µL of total RNA using the TruSeq Stranded Total RNA Library Prep Globin Kit (Illumina Cat. #20020613). Library quantification of the pool was determined using the Library Quantification Kit—Illumina/Universal Kit (KAPA Biosystems, KK4824), which was sequenced on NextSeq 500 High Output flow cells to generate approximately 50 million paired-end 151 bp reads for each sample using NextSeq 500 High Output Kit v2.5 (300 cycles) (Illumina, Cat. #20024908).

**Fig 1:**
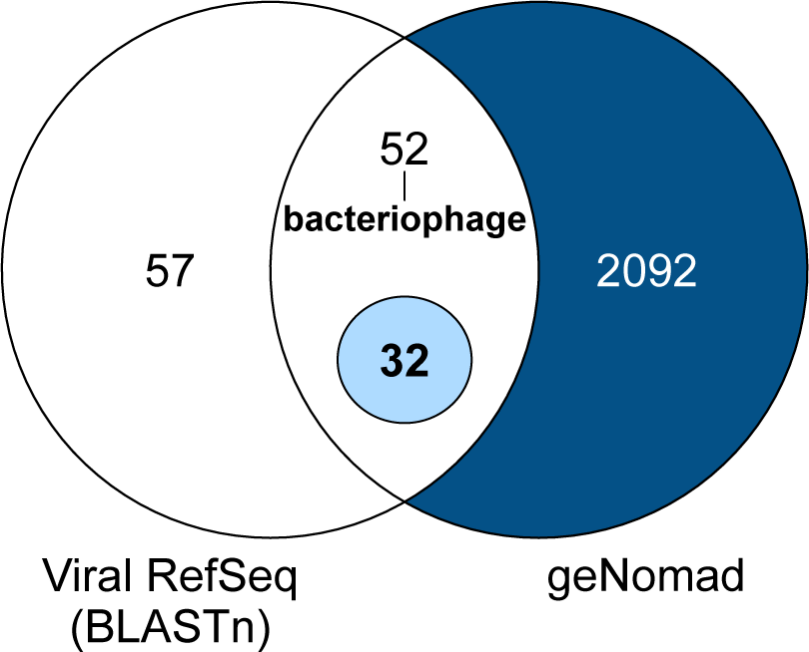
Summary of viral contig annotation. Venn diagram of BLASTn-based and geNomad-based viral taxonomy annotations for assembled contigs (≥1,000 bps).

For each sample, we QCed reads using FaQCs v2.09 (3), then mapped them against the *Felis catus* reference genome (GCF_018350175.1) using Bowtie2 v2.5.2 (4). The unmapped reads were *de novo* assembled using MEGAHIT v1.2.9 (5), creating 559,288 contigs. We selected 45,368 contigs of 1 kb or larger and processed them through geNomad v1.1.0 *end-to-end* mode (6), which classified 2,176 contigs as viruses. We also compared these 45,368 contigs against NCBI’s Viral RefSeq database (accessed 9 May 2024), resulting in 141 contigs with significant BLAST hits to viruses (percent identity >80%; linear coverage >0.5). Eighty-four of these were also classified as viruses by geNomad, including 32 non-bacteriophages (Fig. 1). We focused on recovering longer consensus genomes from 12 samples that contained the 32 contigs by mapping reads to their top RefSeq hits using minimap2 v2.24 (7) in EDGE Bioinformatics (8). If more than 50% of a reference genome was covered and the recovered consensus genome had less than 50% ambiguous bases, the genome was deposited in GenBank and was taxonomically classified by performing a BLASTn search against the entire GenBank database (accessed 29 May 2025).

We report six consensus genomes representing two viral species, including one cat pathogen (felid alphaherpesvirus 1) and one non-pathogenic cat virus (feline foamy virus [FFV]) (Table 1). Felid alphaherpesvirus 1 is a highly contagious pathogen of domestic cats and wild felids and causes upper respiratory tract disease (9). Among the five FFVs, they were 94.4%–100% similar to each other. This study offers an overview of viral diversity in urban domestic cats, providing viral genome sequences, including one cat pathogen, as a resource for future feline virome studies.

**TABLE 1:** Summary of read mapping to NCBI RefSeq references for cat samples from which viral contigs were found^*a*^

Sample ID	Sample type	RefSeq reference for consensus genome	RefSeq reference accession	Length of reference (bp)	Base coverage to reference	NCBI accession of consensus genome	Top hit in GenBank	Accession	BLAST % identity
006	Throat	Felid alphaherpesvirus 1	GCF_000885455.1	135,797	92.6%	PV188038	Felid alphaherpesvirus 1	OQ756207.1	99.99%
029	Rectal	Feline foamy virus	GCF_003047975.1	11,700	78.8%	PV188042	Feline foamy virus	NC_039242.1	97.36%
030	Throat	Feline foamy virus	GCF_003047975.1	11,700	76.9%	PV595280	Feline foamy virus	NC_039242.1	95.56%
033	Rectal	Feline foamy virus	GCF_003047975.1	11,700	78.7%	PV188039	Feline foamy virus	MW389244.1	98.03%
042	Throat	Feline foamy virus	GCF_003047975.1	11,700	70.6%	PV188040	Feline foamy virus	MH633425.1	98.34%
053	Rectal	Feline foamy virus	GCF_003047975.1	11,700	78.8%	PV188041	Feline foamy virus	MW389244.1	97.90%

^*a*^ Consensus genomes containing fewer than 50% Ns were uploaded to NCBI. We BLASTed the consensus genomes and provided the closest BLASTn hit and percent identity.

## Data Availability

The metagenomic sequencing data are available in NCBI’s Sequence Read Archive (SRA) under BioProject accession number PRJNA1052668. The individual NCBI accession numbers assigned to 32 viral contigs can also be found in the BioProject. The accession numbers of the six consensus genomes are listed in Table 1.
